# The role of HDAC6 in enhancing macrophage autophagy via the autophagolysosomal pathway to alleviate legionella pneumophila-induced pneumonia

**DOI:** 10.1080/21505594.2024.2327096

**Published:** 2024-03-11

**Authors:** Minjia Chen, Xiuqin Cao, Ronghui Zheng, Haixia Chen, Ruixia He, Hao Zhou, Zhiwei Yang

**Affiliations:** a Department of Pathogenic Biology and Medical Immunology, School of Basic Medicine, Ningxia Medical University, Yinchuan, China; b Key Laboratory of Fertility Preservation and Maintenance, Ministry of Education, School of Basic Medicine, Ningxia Medical University, Yinchuan, China

**Keywords:** Histone deacetylase 6 (HDAC6), autophagy, *Legionella pneumophila*, autophagyolysosomal pathway, macrophages

## Abstract

*Legionella pneumophila* (*L. pneumophila*) is a prevalent pathogenic bacterium responsible for significant global health concerns. Nonetheless, the precise pathogenic mechanisms of *L. pneumophila* have still remained elusive. Autophagy, a direct cellular response to *L. pneumophila* infection and other pathogens, involves the recognition and degradation of these invaders in lysosomes. Histone deacetylase 6 (HDAC6), a distinctive member of the histone deacetylase family, plays a multifaceted role in autophagy regulation. This study aimed to investigate the role of HDAC6 in macrophage autophagy via the autophagolysosomal pathway, leading to alleviate *L. pneumophila*-induced pneumonia. The results revealed a substantial upregulation of HDAC6 expression level in murine lung tissues infected by *L. pneumophila*. Notably, mice lacking HDAC6 exhibited a protective response against *L. pneumophila*-induced pulmonary tissue inflammation, which was characterized by the reduced bacterial load and diminished release of pro-inflammatory cytokines. Transcriptomic analysis has shed light on the regulatory role of HDAC6 in *L. pneumophila* infection in mice, particularly through the autophagy pathway of macrophages. Validation using *L. pneumophila*-induced macrophages from mice with HDAC6 gene knockout demonstrated a decrease in cellular bacterial load, activation of the autophagolysosomal pathway, and enhancement of cellular autophagic flux. In summary, the findings indicated that HDAC6 knockout could lead to the upregulation of p-ULK1 expression level, promoting the autophagy-lysosomal pathway, increasing autophagic flux, and ultimately strengthening the bactericidal capacity of macrophages. This contributes to the alleviation of *L. pneumophila*-induced pneumonia.

## GRAPHICAL ABSTRACT

**Figure:**
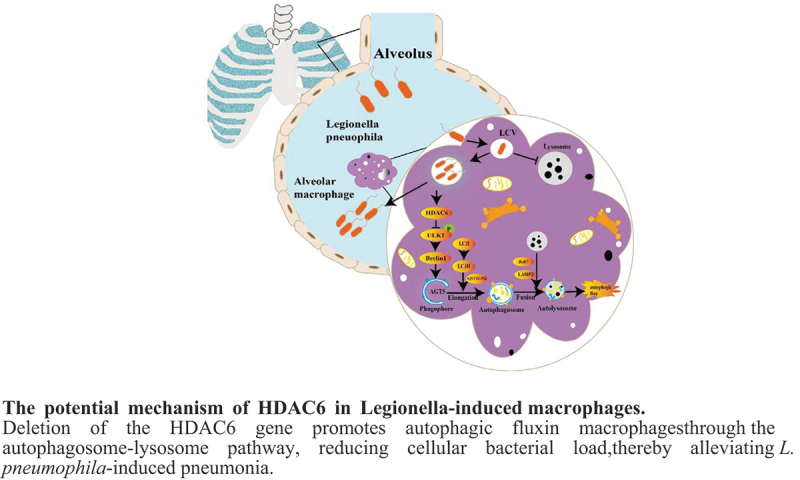

## Introduction

*Legionella pneumophila* (*L. pneumophila*) is a Gram-negative bacterium commonly found in the natural environment. It is an intracellular parasite and can cause Legionnaires’ disease (LD) when humans inhale aerosols containing Legionella bacteria [1,2]. LD is typically classified as community-acquired (CALD), travel-associated (TALD), or healthcare-associated (HALD). Travel-associated LD and ship-associated events are recurring incidents. Cases associated with hotels are frequently connected to hotel cooling towers and potable water systems, while ship-associated cases are most commonly linked to hot tubs [3]. Symptoms of LD often resemble those of the flu, with patients experiencing high fever, chills, cough, chest pain, muscle pain, and headache [4]. This makes accurate diagnosis challenging. The death rate for severe pneumonia caused by *L. pneumophila* typically falls within the range of 5–10%, while it can reach as high as 40–80% in untreated immunosuppressed patients [5]. *L pneumophila* is the predominant strain associated with LD. This bacterium employs the type IV secretion system (T4SS) to release over 300 different types of effector proteins into the host cell’s cytoplasm [6]. These proteins disrupt normal transport pathways within the cell, preventing the fusion of phagosomes and lysosomes and leading to the formation of a Legionella-containing vacuole (LCV) from endoplasmic reticulum vesicles. This LCV contains Legionella bacteria and can inhibit lysosomal degradation and digestive functions [7]. While understanding of *L. pneumophila* has grown, there are still gaps in knowledge regarding the precise mechanisms of infection, transmission, and bacterial replication within host cells. This limited knowledge poses challenges for the development of effective clinical diagnosis, prevention, and treatment strategies. Further exploration of the pathogenic mechanism of *L. pneumophila* can provide the groundwork for more effective preventive measures and clinical diagnosis.

It is currently recognized that autophagy serves as a direct cellular response to infections by various pathogens, including *L. pneumophila*, by facilitating the recognition and degradation of these intruders within lysosomes [8]. Existing evidence has indicated that *L. pneumophila* employs multiple mechanisms to inhibit autophagy, involving the regulation of the Legionella effector RavZ and the interaction with autophagy-related proteins, utilizing autophagy inhibition as a protective measure [9]. The effector protein LegS2 has been reported to suppress starvation-induced autophagy and enhance NF-kB activation by modulating host sphingosine metabolism [10]. Conversely, LegA9 promotes the clearance of *L. pneumophila* in macrophages by upregulating autophagy in bone marrow-derived macrophages (BMDMs) [11]. Autophagy is a lysosomal degradation pathway that maintains cellular homoeostasis by sequestering cellular components and delivering them to lysosomes for degradation when responding to nutrient scarcity and stress [12]. The process of autophagy is characterized by the following sequential stages: (a) the initiation of phagophore formation or nucleation; (b) the conjugation of Atg5 with Atg12, interaction with Atg16L, and the formation of a multi-unit structure at the phagophore; (c) the processing and insertion of LC3 into the expanding phagophore membrane; (d) the capture of random or selective targets for degradation; and (e) the fusion of the autophagosome with the lysosome, followed by proteolytic degradation of engulfed molecules by lysosomal proteases [13]. This process involves the recruitment of specific autophagy-related proteins (ATGs) to form double-membrane vesicles, which encapsulate cellular contents, and subsequently directs them to lysosomes for breakdown [14]. Therefore, the activation of autophagy plays a role in phagocytosing intracellular invaders, as it facilitates the formation of autophagosomes, which can sequester and deliver these invaders to lysosomes for degradation, contributing to their clearance [15]. Disruptions in this clearance process have been found to be associated with various pathologies, including tumorigenesis, metabolic disorders, lung diseases, and infectious diseases [16].

HDAC6 is one of the 18 mammalian deacetylases, belonging to class IIb, and it is primarily localized in the cytoplasm [17]. Notably, HDAC6 is distinguished by its possession of two functional catalytic domains, CD1 and CD2, as well as a zinc finger domain known as ZnF-UBP. Its pivotal role extends to the regulation of a spectrum of biological processes, including gene expression, cell motility, immune responses, and the degradation of misfolded proteins. Additionally, HDAC6 regulates cell migration, chemotaxis, motility and autophagy by deacetylating a-tubulin, cortical protein and HSP90 [18,19]. Due to its multifaceted functions, HDAC6 plays an indispensable role in diverse physiological and pathological processes.

Importantly, HDAC6 exerts regulatory control over various facets of autophagy. This influence encompasses the post-translational modification (PTM) of autophagy-related transcription factors [18,20], participation in the clearance of pathogens via the autophagy pathway [21,22], as well as the facilitation of autophagosome transport and degradation [23]. In addition, HDAC6’s capacity extends to acetylating TFEB, a pivotal factor governing autophagosome-lysosomal fusion. This modification prompts TFEB’s translocation to the nucleus, thereby enhancing autophagosome-lysosome fusion [24]. HDAC6 significantly interacts with cortical proteins, playing a central role in the fusion of autophagosomes and lysosomes during autophagy clearance [25]. When Listeria invades macrophages, macrophages regulate the fusion of phagosomes and lysosomes and the autophagy of cells through the deacetylation function of HDAC6 to remove intracellular bacteria [26]. However, the precise role of HDAC6 in mediating the invasion of macrophages by *L. pneumophila* remains elusive.

The present study aimed to investigate the role of HDAC6 knockout in macrophage autophagy and its subsequent effects on mitigating pneumonia induced by *L. pneumophila*. Therefore, in this study, HDAC6 conditional gene knockout mice were utilized. The knockout of the HDAC6 gene in macrophages enhanced autophagy by activating the autophagy-lysosome pathway, resulting in the inhibition of bacterial proliferation within cells and reducing susceptibility to *L. pneumophila* in mice. The findings may promote the understanding of the molecular mechanisms underlying this process, providing insights into potential therapeutic strategies for LD.

## Materials and methods

### Ethical statement

All animal experiments were approved by the Animal Ethics Committee of Ningxia Medical University (Yinchuan, China; Approval No. 2020–334), and they were undertaken in accordance with the guidelines of the China Animal Welfare Commission.

### Chemical reagents and antibodies

In this study, 4% formaldehyde (P1110), M-SCF (P00085), and activated charcoal yeast extract agar (CYE) (LA7680) were purchased from Beijing Solarbio Science & Technology Co., Ltd. (Beijing, China). Sudan Red (BSBA-4027) and DAB chromogenic solution (ZLI-9017) were purchased from Beijing Zhongshan Jinqiao Biotechnology Co., Ltd. (Beijing, China). MPO antibody (ab208670), *L. pneumophila* antibody (ab20943), and p-ULK1 antibody (ab229909) were obtained from Abcam (Cambridge, UK). β-actin antibody (3700S), HDAC6 antibody (D21B10), SQSTM1/P62 antibody (23214S), and Beclin1 antibody (3495S) were purchased from Cell Signaling Technology (Danvers, MA, USA). LC3B antibody (AF5225), ATG5 antibody (AF2269), ULK1 antibody (AF8307), Rab7 antibody (AF2458), and LAMP2 antibody (AF1036) were purchased from Shanghai Beyotime Biotechnology Co., Ltd. (Shanghai, China). ELISA kits were purchased from Shanghai Mlbio Biotechnology Co., Ltd. (Shanghai, China).

### Animals

Male C57BL/6J mice (age, 8–10-week-old) were purchased from the Animal Experimental Center of Ningxia Medical University. C57BL/6J HDAC6^−/−^ mice were purchased from Syagen Co., Ltd. headquartered in Suzhou, China (KOAIB210311RT2). Animals were reared under specific pathogen-free conditions. All animal experiments were performed according to the protocol approved by the Animal Protection and Use Committee of Ningxia Medical University.

### Bacterial culture

*L*. *pneumophila* (cat no. 35133; American Type Culture Collection, Rockville, MD, USA) was cultured in a xylose yeast extract (CYE) agar plate at 37°C for 3 d, followed by inoculation in a 10 mL CYE liquid medium for 16 h. Then, the concentration was measured using a turbidimeter.

### Mouse infection model

Mice were anesthetized by intraperitoneal injection of a 5% aqueous solution of chloral hydrate (400 mg/kg). The experimental group was inoculated with 30 μL of *L. pneumophila* suspension (1 × 10^7^ CFUs/mouse) using the intranasal instillation method. In the control group (or D0), 30 μL of normal saline was administered nasally. Subsequently, the mice’s body weight and disease symptoms (fluffy fur, hunched appearance, and alertness) were monitored daily.

### Bronchoalveolar lavage fluid (BALF) collection and calculation of bacterial load in the lung

BALF was collected on days 0, 1, 3, and 5 after *L. pneumophila* inoculation. Under anaesthesia with 3% isoflurane, the right lung hilum was occluded using haemostatic forceps. Subsequently, 2 mL of pre-cooled normal saline was slowly infused via a blunt puncture needle for left bronchoalveolar lavage (BAL). The lavage procedure was conducted over a period of 3 min. Following two rounds of mixing, the BALF was subjected to centrifugation at 12,000 g and 4°C for 10 min. The resulting supernatant was collected for the quantification of cytokine concentrations. The lung lobes were dissected and immersed in 5 mL of sterile water. Bacterial colony-forming units (CFUs) in the lung tissue were determined by homogenizing the tissue using a homogenizer, followed by plating the lung tissue homogenate on CYE agar plates at an appropriate dilution. The bacterial load was assessed by counting the number of bacterial colonies that formed in the lungs.

### Pulmonary wet/dry (W/D) ratio

The freshly harvested right lung was rinsed and weighed to determine its initial wet weight. Subsequently, the lung tissue was desiccated in a constant-temperature oven set at 68°C for 48 h. The pulmonary W/D ratio was calculated as an indicator of pulmonary oedema.

### Pulmonary histology

The lung tissue was rinsed with PBS and fixed in 4% paraformaldehyde for 24 h. Subsequently, the lung tissue was progressively dehydrated with increasing concentrations of ethanol, embedded into paraffin, sliced, and stained with haematoxylin-eosin (HE).

### Immunohistochemistry

The lung tissue was fixed in 4% paraformaldehyde and incubated at room temperature for 24 h. Paraffin-embedded tissue sections were then subjected to a dewaxing process to facilitate antigen retrieval. To block endogenous peroxidase activity, hydrogen peroxide (3%) was applied. Each tissue sample was subsequently incubated overnight with anti-LC3B antibody (dilution 1:200) and MPO antibody (dilution, 1:400) at 4°C. Following washing, a horseradish peroxidase (HRP)-conjugated secondary antibody (dilution, 1:1000) was added to the samples and incubated at 37°C for 30 min. After another round of washing, freshly prepared 3,3-diaminobenzidine (DAB) chromogenic solution was utilized. The cell nuclei were counterstained with haematoxylin for 3 min. Subsequently, the tissue slides were sealed. Under light microscopy, DAB-positive staining appeared brownish-yellow, while the nuclei were stained blue. The experiments were conducted using a microscope (BX41; Olympus, Tokyo, Japan), and image analysis was carried out via ImageJ software.

### *Cell preparation and* in vitro *infection model*

BMDMs, from the first-generation culture of bone marrow derived macrophages of mice [27], were utilized. The macrophages were cultured in the complete DMEM, which included 20 ng/mL of recombinant mouse M-CSF (Beijing Solarbio Science & Technology Co., Ltd.), 10% foetal bovine serum (HyClone, Logan, UT, USA), and 100 U/mL penicillin-streptomycin, for 7 days, with a change of medium every 3 days. *L. pneumophila* (at a multiplicity of infection (MOI = 10)) was applied to the antibiotic-free cell culture. After 1 h, clarithromycin (concentration, 8 μg/mL) was added, and the co-culture of macrophages and *L. pneumophila* was maintained for 30 min. Subsequently, the cells were washed with PBS to eliminate clarithromycin and any unphagocytosed bacteria. Fresh medium was then added, and the cultures were maintained until the designated time point.

### RNA extraction and quantitative RT-PCR

Total RNA was extracted from lung tissue homogenates or cells using the NucleoSpin RNA Extraction kit (D6943–02; OMEGA, Brea, CA, USA). Subsequently, cDNA was synthesized following the instructions provided in the PrimeScript RT Reagent kit (RR047A; Takara, Shiga, Japan). Each step of the procedure strictly adhered to the manufacturer’s protocols. Detailed primer sequences can be found in Table 1.

**Table 1.** PCR primers of *L. pneumophila* used for the analysis of gene expression.

Primers	Sequences (5′ → 3′)	Tm (°C)
p62-Forward	TGGGGACTTGGTTGCCTTTT	57.4
p62-Reverse	ATCACATTGGGGTGCACCAT	57.3
Beclin1-Forward	TAATAGCTTCACTCTGATCGGG	53.5
Beclin1-Reverse	CAAACAGCGTTTGTAGTTCTGA	52.9
LC3B-Forward	CCACCAAGATCCCAGTGATTAT	54.1
LC3B-Reverse	TGATTATCTTGATGAGCTCGCT	53.1
HDAC6-Forward	TGCCAAGCGACCTGTGACAATC	53.2
HDAC6-Reverse	GTGGTGGACATAAGCCTGACAGAC	53.7
IL-1β-Forward	TGCCACCTTTTGACAGTGATG	55.9
IL-1β-Reverse	ATGTGCTGCTGCGAGATTTG	56.6
IL-6-Forward	TCCTACCCCAATTTCCAATGCT	56.3
IL-6-Reverse	TGGTCTTGGTCCTTAGCCAC	56.3
IL-10-Forward	ATGCTGCCTGCTCTTACTGACTG	54.7
IL-10-Reverse	CAGGACTACAAGGACGACGATGAC	55

### Western blot analysis

Lung tissue and cells were homogenized in RIPA lysis buffer (P0013B; Beyotime, Shanghai, China). After centrifugation at 12,000 × g for 10 min at 4°C, the supernatant was collected, and the protein concentration was determined using the Beyotime Protein Assay kit (P0010S). Equal amounts of proteins were subsequently separated via sodium dodecyl-sulphate-polyacrylamide gel electrophoresis (SDS-PAGE) and transferred onto polyvinylidene difluoride (PVDF) membranes (Millipore, New York, NY, USA). The membranes were then blocked with 5% skimmed milk, followed by incubation with the primary antibody overnight at 4°C. Following this, the membranes underwent thrice washing with TBST, and the HRP-labelled secondary antibody (1:2000, A0208, Beyotime) was incubated at room temperature for 1 h. Protein bands were visualized using an enhanced chemiluminescence (ECL) solution (A38554; Thermo Fisher Scientific, Waltham, MA, USA) and subsequently quantified using ImageJ software.

### Immunofluorescence

Macrophages were fixed in 4% paraformaldehyde for 15 min and permeabilized with 0.3% Triton X-100 for 10 min. The cells were thrice washed with PBS, blocked with 5% BSA, and the primary antibody was used for incubation at 4°C overnight. After washing with PBS, incubation with horseradish peroxidase (HRP)-labelled secondary antibody, 1:200, A0516, Beyotime) was carried out at 37°C for 1 h, and the nucleus was stained with DAPI (P0131, Beyotime). The samples were sealed with glycerine, and a fluorescence microscope (DMI3000; Leica, Munich, Germany) was employed for microscopic observation. Image analysis was carried out using ImageJ software.

### Transmission electron microscopy

The cells were exposed to *L. pneumophila* for 6 h, after which they were collected. These cells were fixed overnight at 4°C in 2.5% glutaraldehyde (pH 7.3 ~ 7.4) and were then treated with 1% osmium tetroxide for 2 h. The specimens underwent dehydration using a gradient of ethanol and propylene oxide, followed by embedding, sectioning into 50 nm sections, and staining with 3% uranyl acetate and lead citrate. Subsequently, the images were captured using a transmission electron microscope (HT7800; Hitachi, Tokyo, Japan).

### MRFP-GFP-LC3 spot assay

The experiment involved culturing cells in 24-well plates and transfecting them with mRFP-GFP-LC3 adenovirus from Han Bio Technology (Shanghai, China) for 6 h. After replacing the culture medium with a fresh medium for 24 h, *L. pneumophila* (MOI = 10) was incubated for 6 h, followed by examination using a confocal microscope (A1HD25; Nikon, Tokyo, Japan).

### Enzyme-linked immunosorbent assay (ELISA)

According to the manufacturer’s instructions, specific ELISA kits were utilized to quantify the levels of IL-1β, IL-6, and IL-10 in BALF samples. The optical density of each hole was measured at 450 nm using a Micro-board reader (3001, Thermo Fisher Scientific).

### Transcriptome sequencing

The macrophages, derived from bone marrow, were sent to BGI-Shenzhen Corporation (Shenzhen, China) for transcriptome sequencing. Total RNA was extracted using TRIzol reagent, and the isolated RNA came from three independent replicates of transfected samples. RNA concentration and quality were assessed by Agilent 2100 Bioanalyzer (Agilent Technologies, Palo Alto, Calif.). The libraries were sequenced on an Illumina platform, generating 150 bp paired-end reads. Post-sequencing, raw data went through stringent quality control, including adapter trimming, low-quality read elimination, and sequencing artefact filtering to ensure maximum data quality. Cleaned reads were then aligned with the reference genome or transcriptome, providing the basis for subsequent analyses.

#### Identification of differentially expressed genes

To exhibit the similarity and diversity between the two groups, DESeq2 software was employed to compare gene expression differences between the control and treatment groups using the negative binomial distribution. The volcano plot in R, generated using the Ggplot2 package, visualizes the relationship between each target. The DEG heatmap is created using the pheatmap package.

#### Enrichment analysis

The GO analysis, using biological processes, cellular components, and molecular functions, was conducted using R software. The results were obtained by running a script on R, which includes “colorspace,” “stringi,” “ggplot2,” “BiocManager,” “clusterProfiler,” and “enrichplot.” The top 20 items were selected and visualized, with a Bonferroni-corrected *p* value of < 0.05 set as the default. The Kyoto Encyclopedia of Genes and Genomes (KEGG) pathway enrichment analysis method is similar to the aforementioned GO analysis.

#### Protein–protein interaction network

Network analysis was employed to understand the interactions among the genes or proteins of interest, such as protein-protein interaction networks (https://string-db.org) or GeneMANIA online database (https://genemania.org/) was used to analyse the hub.

### Statistical analysis

The data were expressed as mean ± standard deviation (SD) and comprised at least three independent biological replicates. Statistical analysis was conducted using SPSS 27.0 software (IBM, Armonk, NY, USA). Survival rates were assessed using the Fisher’s exact test. To compare differences between two groups, the nonparametric Mann-Whitney U test (for abnormally distributed data) or Student’s t-test (for normally distributed data) was employed. For comparisons involving multiple sets of quantitative data, one-way analysis of variance (ANOVA) was utilized. Paired comparisons were performed using the least significant difference (LSD) test. *p* < 0.05 was considered statistically significant.

## Results

### The expression level of HDAC6 was upregulated in lung tissues infected with *L. pneumophila*

**Figure 1.**
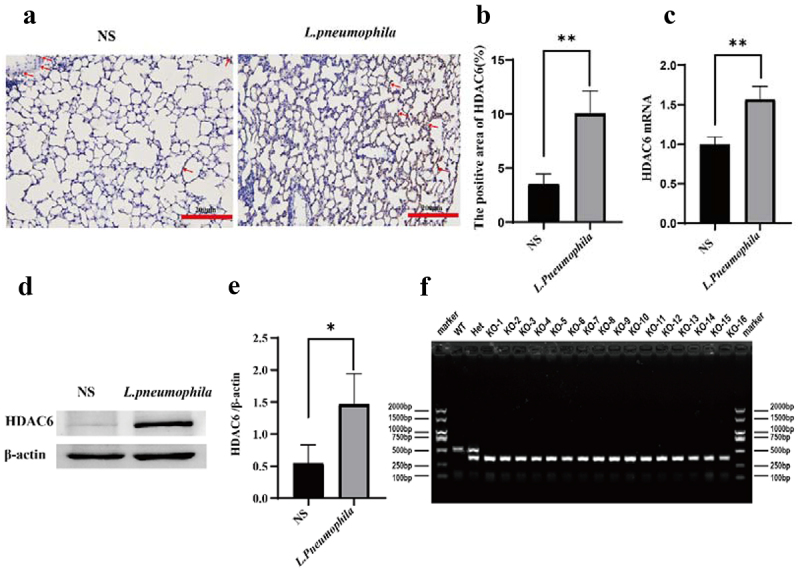
Effects of *L. pneumophila* infection on HDAC6 in lung tissue of mice. (a) Immunohistochemical analysis (200×) reveals HDAC6 expression in the lung tissue of mice infected with *L. pneumophila* after 3days. The brownish-yellow colour indicates positive expression of HDAC6, as indicated by the red arrow. (b) The positive HDAC6 staining in each group was qualified using ImageJ. (c) Impact of *L. pneumophila* infection on HDAC6 mRNA levels in lung tissue. (d) Influence of *L. pneumophila* infection on HDAC6 protein levels in lung tissue. (e) Statistical comparison of HDAC6 protein levels before and after *L. pneumophila* infection. (f) PCR-based genotyping outcomes for select mouse strains: Mice tail RNA gel electrophoresis showing wild-type (WT) with a single 521bp band, heterozygote (Het) with two bands at 380bp and 521bp, and homozygous (KO) with a single 380bp band. NS: normal saline group; *L. pneumophila*: *Legionella pneumophila* group, *n* = 4 mice in each group. Data were presented as mean ± SD. *p < 0.05, **p < 0.01.

Similar to previous studies, immunohistochemical staining revealed that the expression level of HDAC6 in the lung tissue of mice infected with *L. pneumophila* was significantly upregulated (Figure 1(a,b)). RT-qPCR and Western blotting indicated that *L. pneumophila* significantly upregulated the mRNA and protein levels of HDAC6 in lung tissue (Figure 1(c–)). Therefore, HDAC6 plays a crucial role in regulating pulmonary inflammation in mice infected by *L. pneumophila*. This upregulation of HDAC6 May enhance the pro-inflammatory response and contribute to lung tissue damage. Further studies can investigate the specific mechanisms through which HDAC6 influences pathogenicity of *L. pneumonia*, such as its impact on inflammatory signalling pathways and host defence mechanisms. Subsequently, we procured mice with the HDAC6 gene knocked out using CRISPR/Cas9 technology from the company to further investigate its impact on the lung tissue of mice infected with *L. pneumophila*. (Figure 1f).

### Knocking out the HDAC6 enhanced pulmonary protection in mice and reduced the inflammatory response induced by *L. pneumophila*

**Figure 2.**
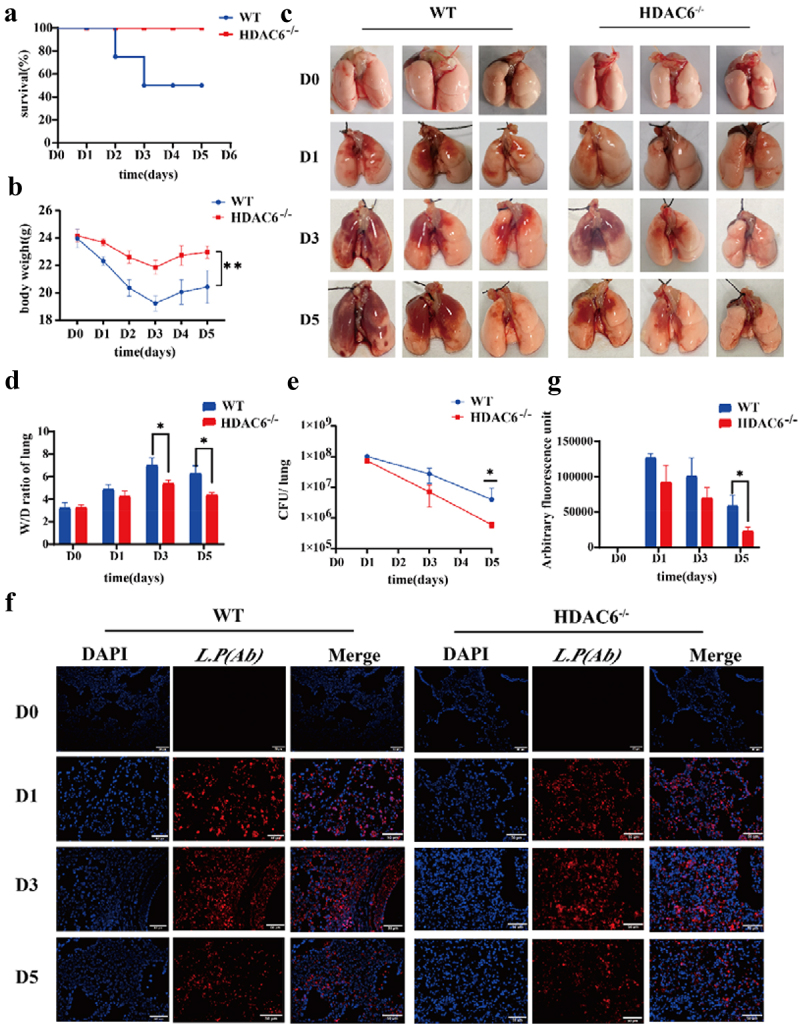
Protective effects of HDAC6 knockout on acute lung injury induced by *L. pneumophila* in a mouse model. (a) Survival rate of mice in the WT group and HDAC6^−/−^ group following *L. pneumophila* infection. (b) Changes in body weight during *L. pneumophila* infection in the WT and HDAC6^−/−^ groups. (c) Representative image of mouse lung. (d) Lung wet/dry ratio in the WT and HDAC6^−/−^ groups after infection by *L. pneumophila*. (e) *L. pneumophila* CFUs in the lungs of chimeras on D1,D3,D5 post-infection in the WT and HDAC6^−/−^ groups. (f) Immunofluorescence labeling for *L.pneumophila* (red) and nucleus (DAPI, blue) in the in the lung tissue of mice showing significantly decreased *L. pneumophila* expression in HDAC6^−/−^ group relative to the WT group. (g) Quantitative analysis of *L. pneumophila* fluorescence in (f). *n* = 4 mice in each group at each time point. Data were expressed as mean ± SD. *p < 0.05, **p < 0.01.

The study of *L. pneumophila* infection model indicated that the body could completely remove *L. pneumophila* within 3–7 days after infection with different concentrations of *L. pneumophila* [28,29]. Therefore, this model (*n* = 4, in each group at each time point) was utilized to assess the changes of WT mice and HDAC6^−/−^mice on days 1, 3, and 5 after infection by *L. pneumophila*. And the control group (D0) received an equal volume of saline solution. Notably, the HDAC6^−/−^ group exhibited enhanced survival rates in mice infected by *L. pneumophila* (Figure 2a) and significantly reduced the weight loss (Figure 2b). As illustrated in Figure 2c, compared with the control group (D0), the lungs of mice infected by *L. pneumophila* generally exhibited various degrees of swelling and congestion. In the WT group, as the infection progressed, the hyperaemic area was enlarged. In contrast, the HDAC6^−/−^ group exhibited a different pattern, with the largest hyperaemia area found on the third day (D3) and a subsequent decrease on the fifth day (D5). Notably, compared with the WT group, the HDAC6^−/−^ group displayed a reduced hyperaemia area at the corresponding time points. Additionally, pulmonary oedema severity was assessed through the pulmonary W/D ratio. The HDAC6^−/−^ group exhibited significantly lower W/D values than the WT group on D3 and D5 (Figure 2d). Furthermore, the bacterial load was evaluated in lung tissue using both bacterial plate test and immunofluorescence. The results indicated a downward trend in bacterial load for both the WT and HDAC6^−/−^ groups throughout the course of infection. However, the HDAC6^−/−^ group consistently displayed significantly lower bacterial loads than the WT group on D3 and D5 (Figures 2(e-g)). These findings suggest that the knockout of HDAC6 enhanced protection against *L. pneumophila* infection in mice.

**Figure 3.**
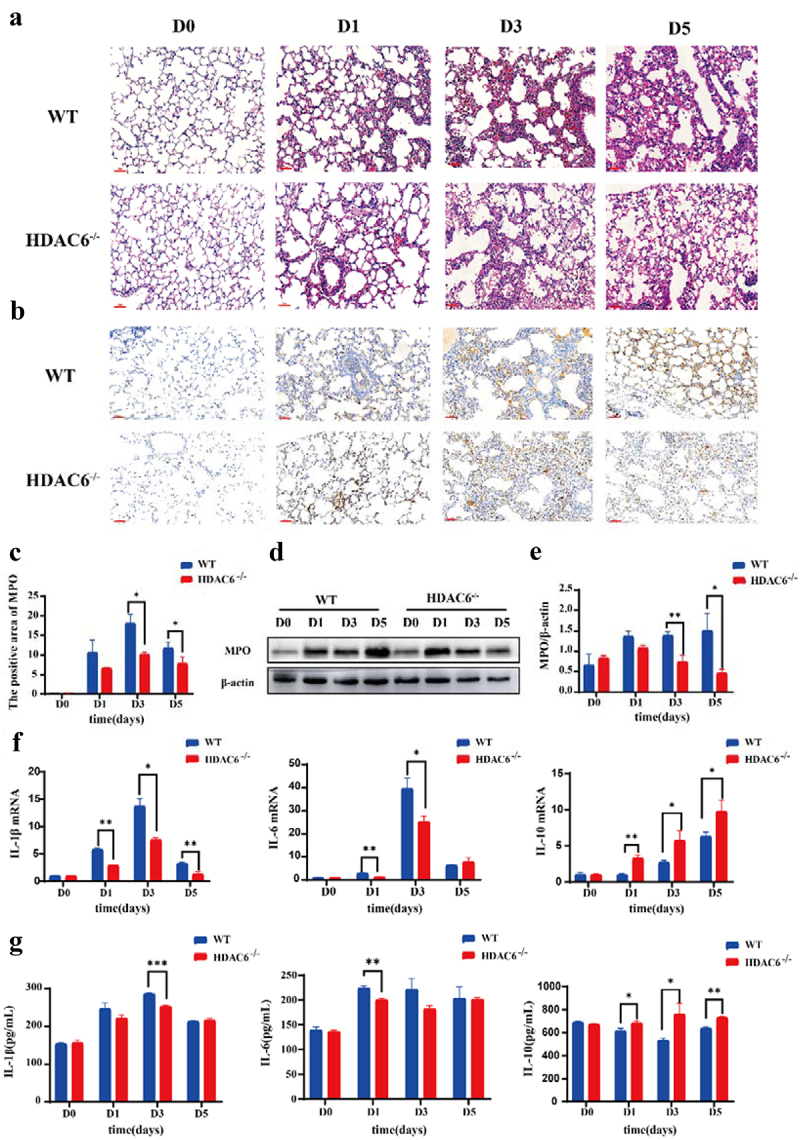
Effects of HDAC6 knockout on lung tissue and expression levels of inflammatory factors in mice infected by *L. pneumophila*. (a) Representative images of haematoxylin-eosin (HE) staining of lung tissue (200×). (b) Immunohistochemical staining was used to detect the expression level of MPO in lung tissue of mice infected by *L. pneumophila* after HDAC6 knockout (200×). The brownish-yellow color indicates positive expression of MPO. (c) The positive MPO staining in each group was qualified using ImageJ. (d and e) Western blotting was employed to detect the expression level of MPO in lung tissue of mice infected by *L. pneumophila* after HDAC6 knockout. (f) The expression levels of inflammatory cytokines (IL-1β, IL-6, and IL-10) in lung tissue of mice infected by *L. pneumophila* after HDAC6 knockout were detected at mRNA level. (g) The secretion levels of inflammatory factors (IL-1β, IL-6, and IL-10) in lung tissue lavage fluid of mice infected by *L. pneumophila* following HDAC6 knockout. The data were expressed as mean ± SD. *p < 0.05, **p < 0.01, ***p < 0.001.

The HE staining showed that the alveolar size of the lung tissue sections in the control group (D0) was basically normal, and no capillary dilation was found. Following *L. pneumophila* infection, both groups exhibited alveolar cavity collapse, presence of neutrophils within the alveolar space, and thickening of the alveolar walls. However, compared with the WT group, the lung tissue sections in the HDAC6^−/−^ group at the corresponding time point exhibited diffuse alveolar collapse, reduced thickening of alveolar space, and neutropenia (Figure 3a). The results of immunohistochemistry revealed that compared with the control group, the MPO activity in lung tissue of mice infected by *L. pneumophila* increased. However, compared with the WT group, the MPO activity in the HDAC6^−/−^ group was significantly reduced at the corresponding time point (Figure 3(b,c)). This result was further verified by Western blot analysis (Figure 3(d,e)). In addition, the effects of HDAC6 knockout on the levels of inflammatory factors (IL-1β, IL-6, and IL-10) in lung tissue were assessed. The findings demonstrated that the HDAC6^−/−^ group exhibited a significant reduction in the mRNA levels and secretion of IL-1β and IL-6 in lung tissue and BALF, while highlighting an increase in the mRNA level and secretion of IL-10 in lung tissue and BALF (Figure 3(f,g)). Collectively, the knockout of HDAC6 could effectively ameliorate lung tissue damage and mitigate the inflammatory response induced by *L. pneumophila* infection in mice.

### Knocking out HDAC6 inhibited intracellular growth of *L. pneumophila* in macrophages

Subsequently, *in vitro* cell experiments were conducted to further verify the impact of HDAC6 knockout on the biological function of macrophages infected by *L. pneumophila*. Initially, murine BMDMs were isolated and characterized [30].

**Figure 4.**
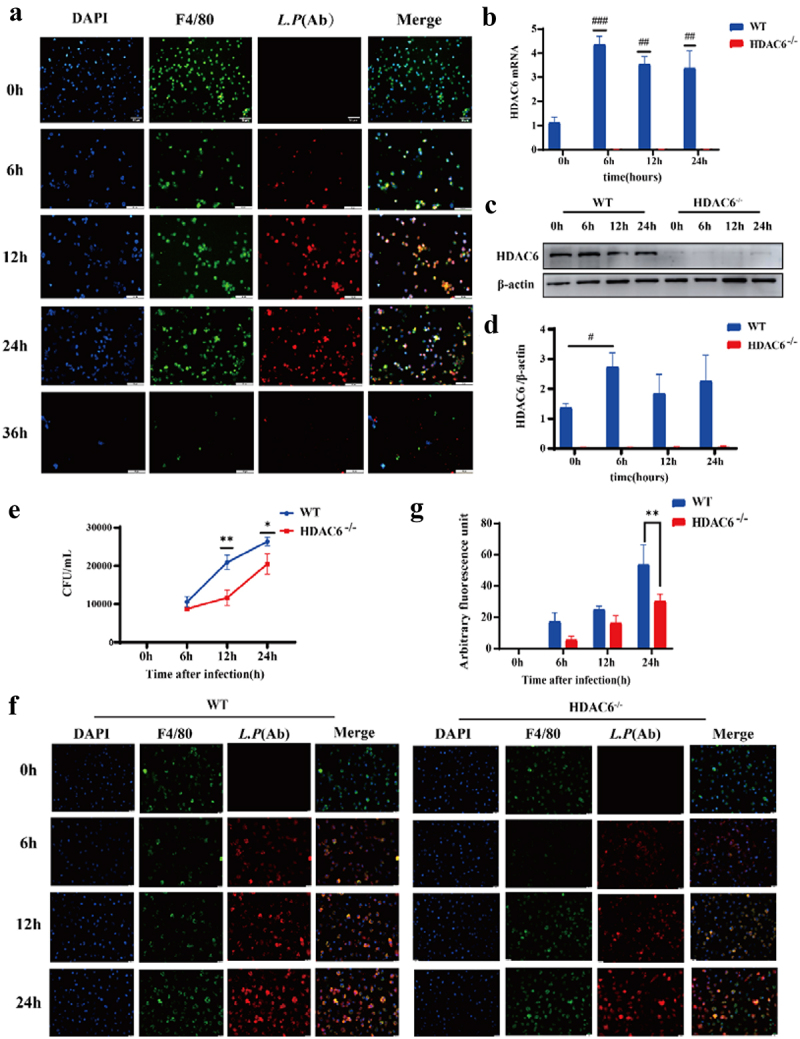
Effects of HDAC6 knockout on the proliferation of *L. pneumophila* in macrophages. (a) Immunofluorescence staining (400×) of *L. pneumophila*-infected macrophages, DAPI: nuclei (blue); F4/80: macrophage surface marker (green); *L.P* (Ab): *L. pneumophila* antibody (red). (b) HDAC6 mRNA levels in macrophages of the WT and HDAC6^−/−^ groups following *L. pneumophila* infection. (c and d) HDAC6 protein expression and statistical analysis in macrophages of the WT and HDAC6^−/−^ groups following *L. pneumophila* infection. (e) Pathogen load expressed as CFU in macrophages of the WT and HDAC6^−/−^ groups following *L. pneumophila* infection. (f) Immunofluorescence labeling for *L. pneumophila*, nucleus, and BMDMs shows a significant decrease in *L. pneumophila* expression in the HDAC6^−/−^ group compared to the WT group. DAPI: nuclei (blue fluorescence); F4/80: macrophage surface marker (green); *L.P* (Ab): *L. pneumophila* antibody (red). (g) Quantitative analysis of *L. pneumophila* fluorescence in (f). Data are expressed as mean ± SD. Intra-group comparison: #*P*<0.05, ##*P*<0.01, ###*P*<0.001; Inter-group comparison: *p < 0.05, **p < 0.01, ***p < 0.001.

Macrophages were subsequently infected by *L. pneumophila* (MOI = 10) to monitor intracellular bacterial growth over time. As depicted in Figure 4a, *L. pneumophila* within macrophages exhibited gradual proliferation over time, reaching its peak at 24 h. After 36 h, cell lysis occurred, indicating the release of intracellular *L. pneumophila*. Consequently, specific time points (0, 6, 12, and 24 h) were selected for investigating bacterial infection in the cells. Figure 4b illustrates that HDAC6 mRNA level significantly increased at 6, 12, and 24 h after *L. pneumophila* infection. However, the protein level of HDAC6 exhibited a significant increase at 6 h (Figure 4(c,d)). Subsequently, *L. pneumophila* growth in both the WT and HDAC6^−/−^ groups was assessed through bacterial plate assay and immunofluorescence. The results indicated that as the duration of *L. pneumophila* infection extended, the number of viable bacteria within macrophages in both the WT and HDAC6^−/−^ groups increased. Nevertheless, at later time points following bacterial infection, the number of viable bacteria in HDAC6^−/−^ macrophages was significantly lower than that in WT macrophages (Figure 4(e-g)). This suggests that the knockout of HDAC6 inhibited the proliferation of *L. pneumophila* in macrophages.

### HDAC6 was involved in the autophagy pathway of macrophages infected with *L. pneumophila* and affected autophagic flux

**Figure 5.**
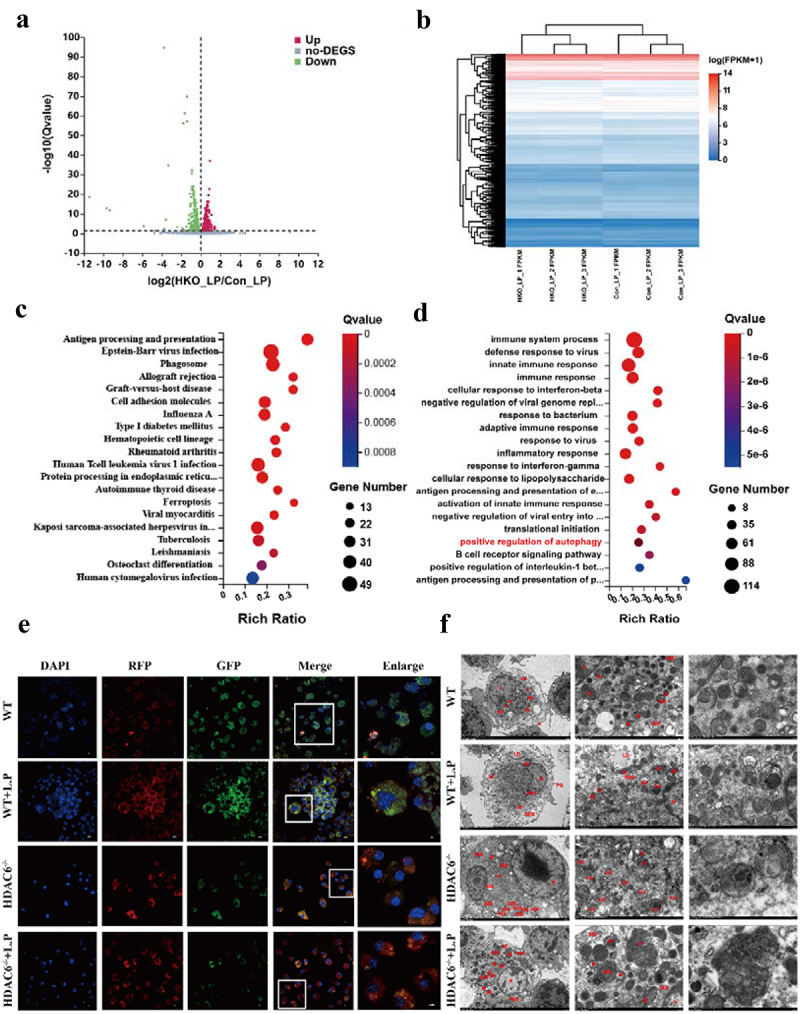
Bioinformatics analysis of HDAC6-mediated signaling pathways in *L. pneumophila*-infected macrophages. (a and b) Volcano plot and heatmap illustrating the expression and clustering of up-regulated and down-regulated genes in HDAC6^−/−^ and WT group macrophages after bacterial addition. (c and d) KEGG and GO bubble diagrams depicting enriched pathways and gene ontology terms. (e) Confocal microscopic images (600×) of cells transfected with mRFP-EGFP-LC3 adenovirus and co-cultured with *L. pneumophila* for 6 h, showing autophagosomes and autolysosomes. (f) Transmission electron microscopy images (1200×, 6000×, 12000×) displaying cellular structures, autophagosomes, and autolysosomes at 6h post-*L. pneumophila* infection. N: nucleus; M: mitochondria; PS: pseudopod; RER: rough endoplasmic reticulum; LD: lipid droplet; Ly: lysosome; ASS: autophagy-lysosome; AP: autophagosomes.

To reveal the important role of HDAC6 in *L. pneumophila*-infected macrophages, the time point of 6 h post-infection was selected and the transcriptome of WT-infected and uninfected macrophages was compared, as well as HDAC6^−/−^ infected and uninfected macrophages, using high-throughput sequencing. For biologically repeated experiments, the negative binomial distribution method was employed to screen out differentially expressed genes (DEGs) with log2 (Fold-Change |>0 and *p* < 0.05). After quantitative analysis and data screening, a total of 1139 DEGs were detected, including 535 upregulated and 604 downregulated DEGs (Figure 5a).

Differential gene cluster analysis involves the clustering of genes that exhibit similar or identical expression patterns, demonstrating their potential participation in shared biological processes, metabolic pathways, or cooperative functional roles (Figure 5b). According to the GO and KEGG pathway enrichment analyses of 1139 DEGs, it was determined that these genes were primarily involved in antigen processing and presentation, phagocytosis, viral infection, and cell adhesion. Furthermore, they were implicated in cellular immune responses, bacterial infection, inflammation, and autophagy, including 19 genes associated with autophagy-related signal transduction pathways (Figure 5(c,d)).

Transfection of mRFP-EGFP-LC3 adenovirus was performed on WT, WT + *L.P*, HDAC6^−/−^, and HDAC6^−/−^ + *L.P* cells to monitor autophagosome formation and autophagosome-lysosome fusion. Autophagosomes appeared as yellow spots, while autolysosomes were observed as red dots (GFP fluorescence is sensitive to acidity). Upon fusion of autophagosomes with lysosomes, GFP fluorescence was quenched, leaving only MRFP (red) fluorescence detectable. As illustrated in Figure 5e, compared with the WT and WT + *L.P* groups, the HDAC6^−/−^ and HDAC6^−/−^ + *L.P* groups displayed a substantial quenching of GFP fluorescence, signifying a reduction in autophagic accumulation and the presence of a stable autophagic flux. This result demonstrated that the knockout of HDAC6 enhanced the autophagic flux in macrophages. Furthermore, TEM observations revealed an increase in autophagic lysosomes and lysosomes in the HDAC6^−/−^ and HDAC6^−/−^ + *L.P* groups in comparison with the WT and WT + *L.P* groups (Figure 5f), aligning with the aforementioned result.

### Knocking out HDAC6 affected the expression levels of autophagy-related factors in murine lung tissues after *L. pneumophila* infection

**Figure 6.**
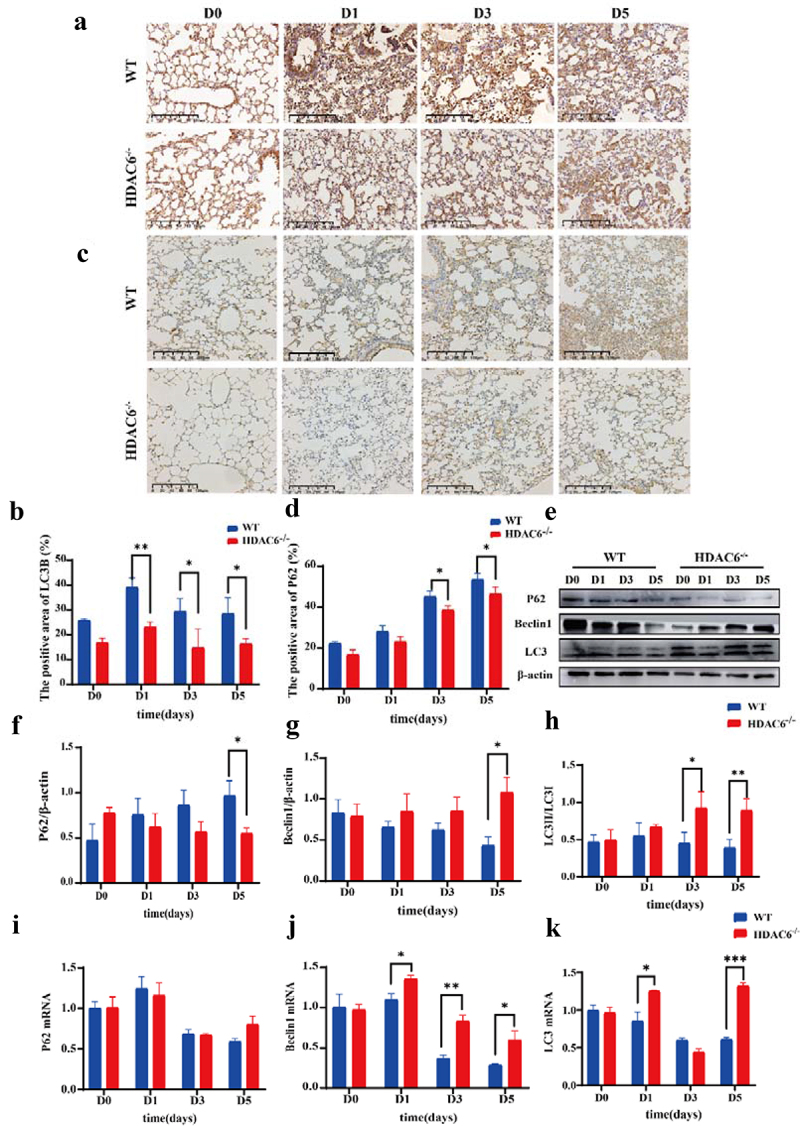
Effects of HDAC6 knockout on autophagy in lung tissue of mice infected by *L.pneumophila*. (a) Immunohistochemical analysis of LC3 protein expression in lung tissue of the WT and HDAC6 knockout groups, the brownish-yellow colour represents positive expression (200×). (b) Statistical results of LC3-positive area in lung tissue using ImageJ software. (c and d) Expression of p62 protein in lung tissue of WT and HDAC6^−/−^ mice infected with *L. pneumophila* and corresponding statistical analysis, the brownish-yellow color represents positive expression (200×). (e) Protein expression levels of Beclin1, p62, and LC3 in lung tissue at different time points following *L. pneumophila* infection. (f-h) Statistical analysis of Beclin1, p62, and LC3 protein expression in lung tissue at different time points. (i-l) mRNA expression levels of Beclin1, p62, and LC3 in lung tissue at different time points following *L. pneumophila* infection. Data were presented as mean ± SD. *p < 0.05, **p < 0.01, ***p < 0.001.

To validate the effects of HDAC6 knockout on autophagy in lung tissue of *L. pneumophila*-infected mice, immunohistochemical staining of LC3B and p62 proteins in lung tissue slices was performed and their expression levels in the WT group and the HDAC6^−/−^ group were determined. The results revealed that the expression levels of LC3B (Figure 6(a,b)) and p62 (Figure 6(c,d)) were significantly elevated in the WT group. Notably, p62 protein is an autophagic substrate, and the increase of p62 expression level may be a sign of blocked autophagic flux. HDAC6 knockout can reduce p62 expression level. Subsequently, Western blotting of autophagy-related proteins (Beclin1, Lc3II/Lc3I ratio, and p62) indicated that in the HDAC6^−/−^ group, the expression level of Beclin1 was upregulated, reaching a significant difference on D5. In contrast, Lc3II/Lc3I ratio was reduced, and the expression level of p62 was also downregulated (Figure 6(e-h)). Additionally, the mRNA levels were measured. The expression level of Beclin1 in the HDAC6^−/−^ group was significantly upregulated at the same time point, and that of LC3B was upregulated on D1 and D5, with no significant differences between the two groups on D3. The mRNA level of p62 did not show significant variations, indicating that the increase in p62 was at post-transcriptional level (Figure 6(i-k)). These results strongly suggested that knocking out HDAC6 could influence the expression levels of autophagy-related factors in murine lung tissues after *L. pneumophila* infection.

### Knockout of HDAC6 promoted macrophage autophagy through the autophagy-lysosomal pathway

**Figure 7.**
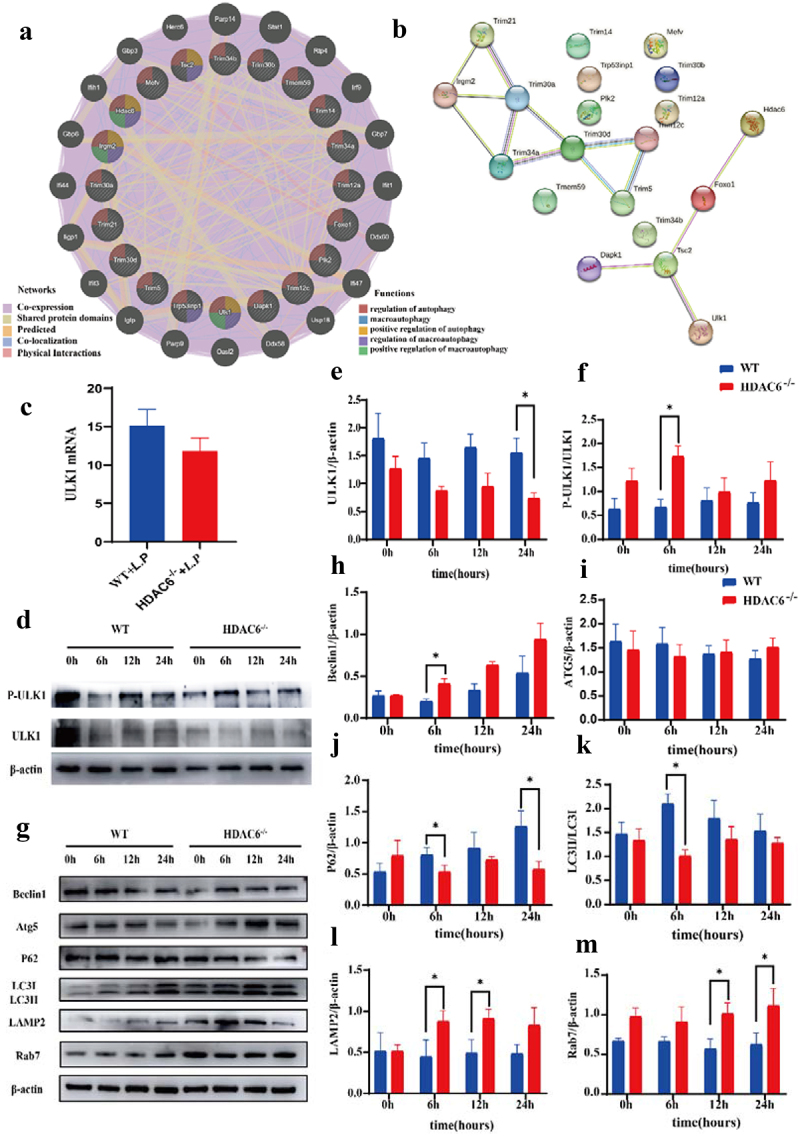
Effects of HDAC6 knockout on autophagy of macrophages infected by *L. pneumophila*. (a) Interaction between autophagy-related genes and HDAC6. (b) Protein-protein interaction (PPI) network showing the interaction between autophagy-related genes and HDAC6 at the protein level. (c) mRNA expression level of ULK1 in WT and HDAC6^−/−^ groups following *L. pneumophila* infection based on transcriptome sequencing. (d) Protein expression levels of ULK1 and p-ULK1 in WT and HDAC6^−/−^ groups after *L. pneumophila* infection. (e and f) Statistical analysis of protein expression of ULK1 and p-ULK1 in cells. (g) Protein expression levels of Beclin1, Atg5, p62, LC3, LAMP2, and Rab7 in cells at different time points after *L. pneumophila* infection. (h-m) Statistical analysis of protein expression for Beclin1, Atg5, p62, LC3, LAMP2, and Rab7 in cells. Data were presented as mean ± SD. *p < 0.05.

To further investigate the role of HDAC6 in the autophagy signalling pathway, the GeneMania was employed to analyse HDAC6 with 19 autophagy-related genes identified by the GO enrichment analysis. ULK1 and Irgm2 were predicted to be closely associated with HDAC6 in terms of their functions (Figure 7a). Subsequently, to explore the relationship between these genes and their involvement in regulatory processes, a PPI network was also constructed (Figure 7b). The analysis of PPI network demonstrated that HDAC6 gene was strongly associated with ULK1, foxo1, and Tsc2 at the protein level. Consequently, HDAC6 could mediate the autophagy signalling pathway by acting on ULK1.

Using transcriptomic data, it was found that ULK1 expression level was downregulated in the HDAC6^−/−^ + *L.P* group, while there was no significant difference (Figure 7c). Therefore, it was attempted to validate this finding at the protein level. Notably, compared with the WT group, ULK1 expression level exhibited a downward trend after *L. pneumophila* infection in the HDAC6^−/−^ group. However, p-ULK1/ULK1 reversed this result, indicating that ULK1 could play a pivotal role at the phosphorylation level (Figure 7(d-f)). Subsequently, the downstream pathway protein of ULK1 was analysed. The expression level of Beclin1 protein increased in the HDAC6^−/−^ group compared with that in the WT group, while there was no significant difference in ATG5 expression level (Figure 7(g-i)). However, compared with the WT group, p62 expression level and LC3II/I ratio were reduced in the HDAC6^−/−^ group after *L. pneumophila* infection (Figure 7(j,k)).

In WT macrophages, some phagocytosed *L. pneumophila* are located in phagosomes, promptly fusing with lysosomes [31]. To further investigate the fusion process between autophagosomes and lysosomes, the expression levels of LAMP2 and Rab7 were identified. The results of Western blot analysis revealed that compared with the WT group, LAMP2 expression level in the HDAC6^−/−^group was significantly upregulated at 6 and 12 h (Figure 7l), and Rab7 expression level was significantly upregulated at 12 and 24 h (Figure 7m). These findings demonstrated that HDAC6 could disrupt the function of the lysosomal membrane and inhibit autophagy after *L. pneumophila* infection in macrophages.

## Discussion

Autophagy is a cellular autonomous defence pathway utilized to limit the replication of microbial pathogens in cells [32]. HDAC6 can regulate autophagy and nucleotide-binding oligomerization domain (NOD)-like receptor family, pyrin domain-containing 3 (NLRP3) inflammasome activation through various mechanisms [33,34]. In the present study, significant upregulation of HDAC6 was found in the lung tissue of mice infected by *L. pneumophila*. Knockout of HDAC6 improved pulmonary inflammation caused by *L. pneumophila* and reduced lung tissue damage. *In vitro*, the deletion of HDAC6 effectively inhibited the proliferation of *L. pneumophila* in macrophages. Furthermore, the deletion of HDAC6 promoted macrophage autophagic flux and increased cellular autophagy through the autophagosome-lysosome pathway. Therefore, this research demonstrated that the knockout of HDAC6 could enhance macrophage autophagy through the autophagosome-lysosome pathway, thereby strengthening the bactericidal effect of macrophages.

This study initially found that HDAC6 expression level was upregulated in the lung tissue of C57BL/6J mice after infection by *L. pneumophila*. In a rat model of chronic obstructive pulmonary disease (COPD), the utilization of HDAC6 inhibitors could prevent the increase in bronchial and pulmonary arterial wall thickness, right ventricular hypertrophy, and pulmonary emphysema caused by cigarette smoke exposure. It can also maintain intact cellular layers and ciliary function in the respiratory mucosa [35,36]. HDAC6 inhibitors not only inhibit the inflammatory signalling pathway and reduce lipopolysaccharide (LPS)-induced acute pulmonary inflammation [37], but also alleviate pulmonary dysfunction and pathological damage caused by acute pulmonary embolism via blocking the AKT/ERK signalling pathway [38]. Moreover, HDAC6 has noticeable functions in inflammatory processes and bacterial infections [39,40]. Silencing of HDAC6 in cell lines not only reduces the secretion of pro-inflammatory cytokine IL-6, but also modulates the expression level of Toll-like receptor (TLR) adaptor myeloid differentiation primary response gene 88 (MyD88). Therefore, the acetylation state of MyD88 not only regulates the NF-kB signalling pathway, but also induces pro-inflammatory cytokines [41]. Conversely, HDAC6 overexpression significantly increased the expression levels of pro-inflammatory cytokines and upregulated the expression level and activity of nicotinamide adenine dinucleotide phosphate (NADPH) oxidase to induce the production of reactive oxygen species (ROS) [42]. In the present study, in mice infected by *L. pneumophila*, alterations in lung tissue structure occurred, accompanied by the release of pro-inflammatory factors, such as IL-1β and IL-6, as well as the anti-inflammatory factor IL-10 in lung tissue and alveolar lavage fluid (Figure 3f,g). However, in the HDAC6 ^−/−^group, pulmonary pathological damage was alleviated at the same time point, reducing the release of pro-inflammatory factors and increasing that of anti-inflammatory factor.

HDAC6 is essential for the acidification of *mycobacterial* phagosomes in macrophages, thereby controlling the intracellular survival of *Mycobacterium* tuberculosis [43]. A previous study demonstrated that knockout of HDAC6 in a cystic fibrosis mouse model resulted in the reduced cytokine level, decreased bacterial load, and limited weight loss [44]. In addition, inhibition of HDAC6 function could restore the intracellular transport of cystic fibroblasts and reduce the activation of NF-kB signalling pathway, indicating a relationship between microtubule regulation and cystic fibrosis [45]. HDAC6-deficient mice exhibited resistance to septic shock induced by intravenous injection of *Listeria* and endotoxin [39,46]. Tubastatin A (HDAC6 inhibitor) can alleviate acute pulmonary injury secondary to lethal sepsis. In addition, it has exhibited to significantly reduce the bacterial load in the spleen and improve the phagocytosis of murine RAW264.7 macrophages *in vitro* [47]. The results of the present study corroborated previous findings, indicating that the knockout of HDAC6 could ameliorate the inflammatory response in mice infected by *L. pneumophila*, resulting in the reduced bacterial load in cells *in vivo*.

Furthermore, the transcriptomic data indicated that knockout of HDAC6 could affect autophagy of macrophages infected by *L. pneumophila*. Previous research demonstrated the direct involvement of *L. pneumophila* effector Lpg2936 in autophagy-related ATG7 enzyme and LC3B-related protein methylation, which is crucial for bacterial replication in cells [48]. In addition, the RavZ effector could regulate autophagic flux by inhibiting the LC3 binding system during Legionella replication [49]. Another study suggested that as long as *L. pneumophila* evades lysosomal fusion, autophagosomes can provide the nutrients required for Legionella replication [50]. In embryonic fibroblasts of mice with HDAC6 gene knockout, lysosomes could be dispersed in the periphery of the cells rather than being concentrated in protein aggregates [51]. This indicates that targeting of lysosomes to autophagic substrates is regulated by HDAC6. A prior study confirmed that S100 calcium-binding protein A11 (S100A11), a calcium-binding protein in liver cells, could inhibit HDAC6, resulting in the upregulation of Forkhead box protein O1 (FOXO1) acetylation, which could enhance its transcriptional activity and activate autophagy [22]. The data of the present study revealed that, in comparison with the WT group, the HDAC6^−/−^ group induced autophagic flux and increased autophagosome-lysosomes in macrophages.

Further mechanistic studies demonstrated that knockout of HDAC6 enhanced autophagy in macrophages induced by *L. pneumophila* through the autophagy-lysosomal pathway. Among the autophagy-related genes, the serine/threonine kinase ULK1 regulated the initiation of autophagy through a complex formed by ATG13, FIP200, and ATG101 [52,53]. Under stress conditions, the activation of ULK1 could not only be induced through AMPK-mediated phosphorylation, but also by mTOR inhibition [54]. However, KAT5/Tip60-mediated ULK1 acetylation is different from mammalian target of rapamycin complex 1 (mTORC1) and AMPK-mediated ULK1 phosphorylation in activating ULK1 [21]. The ULK1 complex activates the BECN1 (Beclin 1)-PIK3C3/Vps34 complex to trigger the autophagy cascade, thereby promoting the formation and expansion of phagocytes [55]. The BECN1-PIK3C3 complex is acetylated by the acetyltransferase EP300/p300 and promotes the interaction between Beclin 1 and the negative regulator RUBCN/Rubcon of PIK3C3 [56,57]. Prior research demonstrated that apigenin, an HDAC inhibitor, promoted autophagic cell death in gastric cancer by increasing ATG5 expression level, a marker of autophagy, as well as the phosphorylation of AMPK and ULK1 [58]. HDAC inhibitors enhance the lethality of niraparib by increasing the activation of ATM-AMPK-ULK1-autophagy and CD95-FADD-caspase 8 pathways [59]. The findings of the present study indicated that HDAC6 modulated the expression level of autophagy-related gene ULK1, leading to the regulation of the autophagy-lysosomal pathway. The knockout of HDAC6 resulted in the decreased ULK1 expression level, increased ULK1 phosphorylation, and upregulated the expression level of downstream Beclin1.

ATG5 is essential for both classical and non-classical autophagy pathways [60]. Under conditions of starvation or rapamycin inhibition, receptor for activated C-kinase 1 (RACK1) binds to ATG5, initiating the formation of autophagosomes [61]. The role of acetylation in the regulation of ATG5 and autophagy function has remained elusive. Previous research indicated that ATG5 could undergo acetylation by EP300 [62]. It was reported that ATG5 could regulate the DNA damage response independently of autophagy [63]. Several studies have demonstrated that p62 could promote the expression level of HDAC6, reduce the acetylation levels of microtubules, and inhibit autophagy in hormone-independent prostate cancer cells [64]. In addition, studies have found that p62 could inhibit the deacetylase activity of HDAC6 to enhance the acetylation of microtubules or cortical proteins and promote autophagic flux [65,66]. Notably, p62 protein is an autophagy substrate that binds to LC3B-II in autophagosomes and degrades after the formation of autolysosomes [67], which is consistent with the conclusion of the present study. The elevated expression level of p62 protein suggests a blockade in autophagic flux. The deacetylation of LC3 has a significant impact on starvation-induced autophagy [68]. Following starvation treatment of HeLa cells, HDAC6-mediated deacetylation of LC3-II enhances autophagic flux, whereas the increased acetylation of LC3-II in HDAC6 siRNA HeLa cells hinders autophagic flux [69].

These studies have elucidated that HDAC6 functions as a deacetylase or scaffold protein to modulate LC3. Given the accumulation of autophagosomes (elevated LC3II/I ratio) induced by *L. pneumophila* infection in the WT group in the present study, it suggests an increase in the number of autophagosomes or inhibition of autolysosome formation due to the impaired fusion between autophagosomes and lysosomes.

Lysosomes may serve as crucial degradation centres in most eukaryotic cells [70]. Proper transport, acidification, and fusion with late autophagosomes are essential for maintaining lysosomes’ normal function [71]. Rab7 plays a multifaceted role in regulating autophagy by marking autophagosome maturation, directing cargo transport along microtubules, and participating in the fusion step with lysosomes [72]. LAMP2 promotes autophagic vacuole maturation by facilitating vesicle fusion events on microtubules [73]. It is also involved in the transport of endosomal/lysosomal cholesterol, and its loss may lead to delay in the transport of early endosomal to lysosomal fluid markers [74]. Consequently, both Rab7 and LAMP2 are crucial for the late-stage fusion of autophagosomes and lysosomes. The results of the present study indicated that when *L. pneumophila* interfered with macrophages in the WT group, lysosomes and autophagosomes failed to fuse correctly, resulting in the accumulation of unfused autophagosomes. Conversely, in the HDAC6^−/−^ group, the expression levels of LAMP2 and Rab7 were upregulated after *L. pneumophila* infection in macrophages (Figure 7 (,)). It indicated that HDAC6 could affect lysosomal function.

*L*. *pneumophila* has evolved multiple mechanisms to evade the immune system and regulate inflammatory responses, including inhibiting phagosome maturation to escape fusion with lysosomes [75], manipulating host immune signalling pathways, and interfering with host cell autophagy processes [75,76]. However, the mechanism by which *L. pneumophila* utilizes HDAC6 to evade the immune system and cause LD remains unclear. The recent discovery reported that *L. pneumophila* encodes an HDAC-like protein (LphD) in THP-1 cells, which synergistically collaborates with a eukaryotic methyltransferase (RomA) by specifically deacetylating H3K14 to facilitate methylation of H3K14 by RomA. Thereby fine-tuning the host cell’s gene expression and fostering bacterial intracellular replication [77]. It indicates that HDAC plays an important role in the immune evasion of *L. pneumophila*. Our research indicates that HDAC6 May play a role in suppressing host cell’s autophagy, further contributing to the bacterium’s intracellular survival. Collectively, *L. pneumophila* has evolved a multifaceted approach to circumvent the host immune system for replication in cells. The details of these interactions, including the potential involvement of HDAC6, continue to be a subject of active scientific investigation, providing insights into possible targets for the future therapeutic interventions.

In conclusion, it was indicated that HDAC6 expression level was upregulated in murine macrophages after *L. pneumophila* infection. Knocking out HDAC6 could promote autophagy through the autophagolysosomal pathway in murine macrophages, thereby enhancing their bactericidal capabilities and alleviating *L. pneumophila*-induced pneumonia. The upregulation of HDAC6 in response to infection suggests a complex interplay between this enzyme and the bacterium. The enhanced inflammatory response and pulmonary tissue damage observed in the presence of elevated HDAC6 level highlight the role of this enzyme in promoting the pathogenicity of *L. pneumophila*. These findings support HDAC6 inhibition as a promising potential therapeutic target for the treatment of LD caused by *L. pneumophila*. In summary, this study expands our understanding of the pathogenic mechanisms of *L. pneumophila* by highlighting the role of HDAC6 knockout in inflammatory responses during infection. Further exploration of the specific signalling pathways and host defence mechanisms affected by HDAC6 knockout could provide new targets for preventive and therapeutic interventions against LD, including the development of specific HDAC6 inhibitors and clinical trials to evaluate their efficacy.

## Data Availability

The authors confirm that the data supporting the findings of this study are available within the article and/or its supplementary materials.
